# Modeling CAF-tumor interactions to overcome therapy resistance

**DOI:** 10.1186/s13046-025-03633-y

**Published:** 2026-01-08

**Authors:** Jakub Gubala, Daniel Benamran, Petros Tsantoulis, Valentin Mieville, Massimo Valerio, Patrycja Nowak-Sliwinska

**Affiliations:** 1 Cancer Systems Pharmacology Group, School of Pharmaceutical Sciences, University of Geneva, Geneva, 1211 Switzerland; 2 Institute of Pharmaceutical Sciences of Western Switzerland, University of Geneva, Geneva, 1211 Switzerland; 3 Translational Research Center in Oncohaematology, Geneva, 1211 Switzerland; 4 Division of Urology, Geneva University Hospitals, Geneva, Switzerland; 5 Department of Oncology, Geneva University Hospitals, Geneva, Switzerland

**Keywords:** Cancer-associated fibroblasts, Clear cell renal cell carcinoma, Combined treatment, The extracellular matrix, Tumor microenvironment

## Abstract

Cancer-associated fibroblasts (CAFs) represent an interesting component of the tumor microenvironment, playing a crucial role in cancer progression, metastasis, and treatment resistance. This review provides a comprehensive overview of identified subtypes across various cancer types, highlighting their inter-tumor heterogeneity, plasticity, and function of the different CAF phenotypes. With a focus on renal cell carcinoma, we reviewed the correlations between CAF markers, disease progression, and treatment outcomes. This approach underlined the existence of CAF-induced drug resistance and therapeutic strategies that can be utilized to overcome this extrinsic resistance. A major focus of this review is the role of CAFs in inducing drug resistance, a phenomenon observed in both *in vivo* and *in vitro* cancer models. We discuss the integration of CAFs in several *in vitro* co-culture systems cultivated in two- and three-dimensional models, as well as their contributions to deciphering CAF-tumor crosstalk. These platforms better recapitulate the tumor microenvironment, reflecting CAF-induced therapeutic resistance. Ultimately, this review underlines the crucial role of CAF-incorporated co-culture systems in advancing drug development, especially in the context of tissue- and subtype-specific CAF targeting.

## Introduction

Cancer prevails as the leading cause of death worldwide. Despite recent advancements in therapeutic strategies, an unmet need for new approaches remains. Frequently, research focuses mostly on cancer cells, overlooking the intricate dynamics of the tumor microenvironment (TME). TME contains various cell types, including fibroblasts, immune cells, pericytes, and endothelial cells (ECs), as well as extracellular matrix (ECM) and signaling molecules. Constant communication between TME and cancer cells can contribute to increased tumor proliferation and invasion, drug resistance, inflammation, cancer cell survival, or immune evasion. Cells within TME are often also described as being responsible for increasing the tumor heterogeneity [1]. Pre-clinical models frequently lack the architectural complexity of the tumor microenvironment and fail to consider the complexity of TME-cancer cell interactions, leading to inaccurate drug screening models [2, 3].

Among various cell types present in the TME, fibroblasts have been of particular interest in recent years. More specifically, highly specialized activated sub-populations called cancer-associated fibroblasts (CAFs). CAFs have been described in various tumor types as one of the key factors in cancer progression [4]. CAFs can originate from multiple cell types, including resident fibroblasts, mesenchymal stem cells, endothelial cells, or cancer cells through epithelial-to-mesenchymal transition (EMT). Activation is mediated by cytokines and growth factors, such as transforming growth factor-beta (TGF-β), fibroblast growth factor (FGF), and platelet-derived growth factor (PDGF). Upon activation, CAFs undergo metabolic and epigenetic changes, altering their secretory profiles. Unlike normal fibroblasts, whose primary role is wound healing and tissue repair, CAFs often adopt a pro-tumorigenic phenotype, promoting tumor cell proliferation, invasion, and metastasis, ultimately contributing to the development of therapy resistance [4, 5].

This pro-tumorigenic effect of some CAF populations is mediated by paracrine (secretion of soluble factors such as cytokines or ECM-remodeling factors, miRNAs, or metabolites) and juxtacrine (direct cell-cell contact) signaling. These interactions are not limited to CAFs and cancer cells, but also extend to other cell types within the TME, e.g., immune or endothelial cells [6–9]. CAFs have been previously shown to create an immunosuppressive environment by blocking natural killer cells activation, reducing cytotoxic T cells function, driving trans-differentiation of macrophages into a tumor-promoting subtype, and enhancing differentiation of T cells into immunosuppressive regulatory T-cells [10]. Moreover, the presence of CAFs has been linked to lymphangiogenesis and lymph node metastasis. Through these interactions, CAFs play a pivotal role in shaping the TME, making them potential therapeutic targets. Therefore, it is important to realize that the presence of CAFs in preclinical models significantly influences drug development and the selection of the lead drug candidates. While the reviews on reconstructing the TME exist, e.g [11].,, the implementation of CAFs in the co-culture models has not been exhaustively addressed.

In this review, we summarize current knowledge on CAFs, emphasizing their biological traits, the mechanisms by which they influence tumor behavior, and their application in *in vitro* models. We also highlight their inter-tumor heterogeneity and differing effects on cancer upon release of similar factors across tumor types. Lastly, we focus on CAF-induced resistance, also observed *in vitro*, which underlines their significance in *in vitro* models designed to assess potential treatment efficacy.

### CAF subpopulations across cancer types

CAF’s phenotype is shaped by numerous factors present within the TME, including interactions with the tumor cells, immune cells, ECM physico-chemical characteristics, or spatial position within the tumor. In recent years, advancements in single-cell sequencing technologies have led to a significant rise in studies aiming to identify and characterize CAF subtypes across tumor types [5, 11–13]. The phenotypes of CAFs can differ across tumor types, but also intratumorally, where various subpopulations can often be identified [5, 11, 12, 14–18]. These subpopulations can have either tumor-promoting or tumor-restraining functions [12]. Even if certain CAF subpopulations share the same names across tumor types, they may exhibit distinct sets of markers that differentiate them into separate clusters. Notably, myofibroblasts (myoCAF, myCAF, CAF_myo_, or matrix CAF [19]), a subpopulation identified in numerous cancers, are frequently associated with matrix remodeling and/or contractile properties. Concerning other subpopulations, while similarities can be observed among myCAFs across different cancers, the markers used to identify them often vary significantly.

While a universal classification system remains elusive, some researchers have used broadly available single-cell RNAseq (scRNA_seq_) databases to classify CAFs across multiple cancer types [20–22]. Luo et al. [22] studied ten solid tumors and identified 8 clusters annotated as fibroblasts, which were further classified into two main groups: (i) normal fibroblasts (NF), which contained two clusters, and (ii) CAFs, which contained three major clusters: myoCAFs, inflammatory CAFs (CAF_infla_ or iCAF), and adipogenic CAFs (CAF_adi_). Another three minor clusters contained endothelial-to-mesenchymal transition CAFs (CAF_EndMT_), peripheral nerve-like CAFs (CAF_PN_), and antigen-presenting CAFs (CAF_ap_).

The major clusters were characterized by canonical markers present on each subtype: ACTA2 on myoCAF, FAP, and/or TGF-β1 on iCAF or CFD for CAF_adi_.

A similar study was performed by Galbo et al. [20], who determined the CAF subclusters based on data from melanoma, head and neck squamous carcinoma, and lung cancer. Apart from one normal fibroblast cluster, the authors identified 5 pan-CAF subtypes: (i) myofibroblast-like CAFs, (ii) desmoplastic-like CAFs, (iii-iv) two types of inflammatory-like CAFs, and (v) proliferating-like CAFs.

Based on breast cancer, colorectal cancer (CRC), liver hepatocellular carcinoma, ovarian cancer, prostate adenocarcinoma, and uterine corpus endometrial carcinoma tissues, Ma et al. [19] described four CAF sub-populations, i.e., iCAF, matrix CAF (mCAF), metabolic CAF (meCAF), and proliferative CAF (pCAF).

Discrepancies in findings between these studies may stem from either methodological differences or inconsistencies in CAFs’ nomenclature. The latter issue could be resolved by implementing a standardized nomenclature based on biomarkers and specialized functions [13]. The authors suggested terms, such as “PDPN^+^Ly6c^+^ immune regulatory CAFs” or “PDPN^+^LRRC15^+^ myofibroblastic CAFs “. This approach could facilitate understanding of the functional roles of these subpopulations and enable distinction between the CAFs, confused by the use of identical names across various cancers. Differences in tumor type likely also contribute to the variations observed between cited classifications. For instance, antigen-presenting (apCAF) cells were present predominantly in pancreatic ductal adenocarcinoma (PDAC) and breast cancer, while vascular CAFs (vCAF) were identified predominantly in cholangiocarcinoma [12, 23]. These CAF subpopulations exhibit significantly distinct characteristics from more common subpopulations, which can be attributed to the heterogeneous origin of CAFs [12].

As outlined by Lavie et al. [13], CAF subpopulations can be described using methods other than single-cell transcriptomics, e.g., flow cytometry, immunofluorescence (IF), or in situ hybridization. While these techniques provide valuable preliminary insights into CAF subpopulations, scRNA_seq_ provides higher levels of complexity and detail. However, standard scRNA_seq_ does not provide information regarding the spatial distribution of identified subpopulations. Hence, multiple studies have integrated scRNA-seq data with spatial findings, using, e.g., spatial transcriptomics [19, 21, 24, 25], multiplex imaging mass cytometry [14], IF, or immunohistochemistry [16, 22, 26].

Chen et al. [12] suggest that CAF subpopulations classification should only be performed after careful functional analysis, as relying exclusively on transcriptomics-based classification might lead to an overinterpretation of the cell phenotypes. For instance, certain markers, such as interleukin 6 (IL-6), known to identify inflammatory CAFs, can also be expressed by myCAFs.

Similarly, certain ECM components typically linked to myCAFs can also be expressed by iCAFs. This further highlights the complexity of CAF classification and suggests that multidimensional approaches are necessary to reliably distinguish these subpopulations.

While the myCAF and iCAF framework provides a valuable model for understanding CAF heterogeneity, it is increasingly recognized that CAFs exist on a functional spectrum that extends beyond this dichotomy. A more comprehensive perspective classifies CAFs based on their contextual roles within the TME, encompassing both tumor-promoting (e.g., through ECM remodeling, immunosuppression, and angiogenic signaling) and tumor-suppressive subsets (e.g., through antigen presentation and tumor-restraining ECM deposition) [27, 28]. This functional plasticity underscores the critical importance of the specific tumor microenvironment in determining CAF behavior.

Interestingly, it has been demonstrated that the CAFs may transition into other subpopulations, specifically into iCAFs and myCAFs [29, 30]. In an *in vitro* PDAC model, this transition into either subpopulation was shown to be driven by competing signaling pathways, i.e., TGF-β promotes myCAF phenotype, while JAK/STAT promotes the iCAF phenotype [30, 31]. This model demonstrated that increased TGF-β signaling or decreased JAK/STAT signaling reduced IL-1R expression, thereby suppressing the iCAF phenotype while promoting myCAF differentiation. Conversely, blocking TGF-β signaling induced a transition to the iCAF phenotype. This finding raises an important question of whether similar phenotypic transformations can be observed *in vivo*, in other types of cancers, between other CAF subpopulations, or whether the *in vivo* phenotype is instead shaped by the diverse origin of the CAFs.

### Reported CAF markers across cancer types

CAFs populations with similar functions can exhibit different sets of markers depending on the tumor type. A comprehensive list of CAF markers is provided in Table 1.

**Table 1:** The summary of the most used CAF biomarkers across various cancers. ccRCC – clear cell renal cell carcinoma, CRC – colorectal cancer, PDAC – pancreatic ductal adenocarcinoma

Name of the marker	abbreviation	Tumor type
α-smooth muscle actin	ACTA2	Breast [11, 18, 28, 32–37], CRC [28, 38–40], Gastric [41], PDAC [11, 42–44], Lung [37, 45, 46], Head and neck [47, 48], Liver [20], Melanoma [41, 49, 50], ovarian [41, 49, 50], Prostate [51], ccRCC [40, 52–55]
Caveolin 1	CAV-1	Breast [11, 32, 37], Gastric [40], Lung [46], Ovarian [49], PDAC [40]
Thy-1 cell surface antigen	CD90/Thy-1	Breast [34], Lung [46], Melanoma [56]
Collagen type XI alpha 1	COL11A1	Breast [57], ccRCC [58, 59], Ovarian [50]
Collagen type XVI alpha 1	COL16A1	ccRCC [40, 52, 58]
Collagen type I alpha 1	COL1A1	Breast [34], CRC [39] Head and neck [48], Liver [47], Melanoma [20, 56], Prostate [51], ccRCC [40, 52, 54, 58]
Collagen type I alpha 2	COL1A2	Breast [34], CRC [38, 39], Prostate [51], ccRCC [58], Melanoma [56]
Collagen type IV alpha 1	COL4A1	Lung [45]
Collagen type VIII alpha 1	COL8A1	Liver [47]
Collagen type X alpha 1	COL10A1	Lung [45]
Collagen type XIV alpha 1	COL14A1	Breast [35]
C-X-C motif chemokine ligand 12/Stromal Derived Factor 1	CXCL12/SDF-1	Breast [18, 33–35], CRC [39], Gastric [41], PDAC [11, 43, 44], Ovarian [41, 49, 50]
Desmin	DES	PDAC [44]
Elastin microfibril interfacer 1	EMILIN1	ccRCC [55, 58]
Fibroblast Activation Protein	FAP	Breast [11, 32, 34, 35], CRC [28, 38, 39], PDAC [11, 42–44], Head and neck [48], Lung [46], Ovarian [49, 50], ccRCC [52–54], Melanoma[56]
Fibronectin	FN1	ccRCC [52]
Fibroblast Specific Protein 1	FSP-1/S100A4	Breast [11, 32, 33], CRC [38], Lung [46], ovarian [49]
Hepatocyte Growth Factor	HGF	Liver [40]
Intercellular Adhesion Molecule 1	ICAM-1	CRC [39]
Interleukin 6	IL-6	Breast [35], CRC [60], PDAC [11, 42–44], Gastric [61], Ovarian [50, 62], Melanoma [56]
Interleukin 8	IL-8	Breast [18], PDAC [11, 43], ccRCC [52]
Lysyl oxydase homolog 1	LOXL1	ccRCC [58]
Leucine rich repeat containing 15	LRRC15	Breast [11, 36], PDAC [63]
Lumican	LUM	CRC [38], ccRCC [58], Melanoma [56]
Major Histocompatibility Complex class II (related genes)	MHC-II (related)	Breast [35], Lung [64], PDAC [11, 22, 42–44], Prostate [51], ccRCC [52]
Matrix metalloproteinase 11	MMP11	PDAC [11, 22, 43], ccRCC [52]
Matrix metalloproteinase 2	MMP2	CRC [38], ccRCC [55]
Matrix metalloproteinase 3	MMP3	Gastric [61], Lung [45], Oral Squamous Cell Carcinoma [37, 65], Melanoma [56]
Matrix metalloproteinase 9	MMP9	Lung [66], Melanoma [56]
Platelet Derived Growth Factor α/β	PDGFA/B	CRC [38]
Platelet Derived Growth Factor Receptor α/β	PDGFRA/B	Breast [11, 16, 28, 32, 40], CRC [40], Lung [46], PDAC [11, 43, 44], Prostate [40], Gastric [41, 61], ovarian [40, 41, 49], ccRCC [55]
Programmed Death Ligand 1	PD-L1	PDAC[67]
Podoplanin	PDPN	Breast [34, 40], CRC [39], Gastric [40], Head and neck [48], Lung [40, 46], Ovarian[49]
Periostin	POSTN	PDAC [43], CRC [40], Gastric [61, 68], Lung [46], Ovarian [40], ccRCC [52], Melanoma [56]
Regulator of G-protein signaling 5	RGS5	Breast [11, 16], Liver [47]
Transgelin	TAGLN	Breast [35], CRC [38, 39], ccRCC [52], Gastric [41, 61, 68], Melanoma [20], Ovarian [41], PDAC [11, 43], Prostate [51]
Transforming Growth Factor β	TGF-β	Breast [11, 18, 33, 35, 36], Oral Squamous Cell [65], Ovarian [50], PDAC [44], ccRCC [54]
Tenascin-C	TNC	Breast [33, 35], CRC [40], Lung [46], Melanoma [56]
Vascular Endothelial Growth Factor	VEGF	Breast [18]
Vascular Cell Adhesion Molecule 1	VCAM-1	ccRCC [55]
Vimentin	VIM	Lung [40, 46], Oral [40], PDAC [44], ccRCC [52]

Functionally, distinct CAF markers have been associated with diverse roles in tumor biology and progression. Their well-documented contributions include ECM remodeling, immunosuppression, promotion of tumor growth, promotion of metastasis, and promotion of angiogenesis [5, 28, 69].

**Fig. 1:**
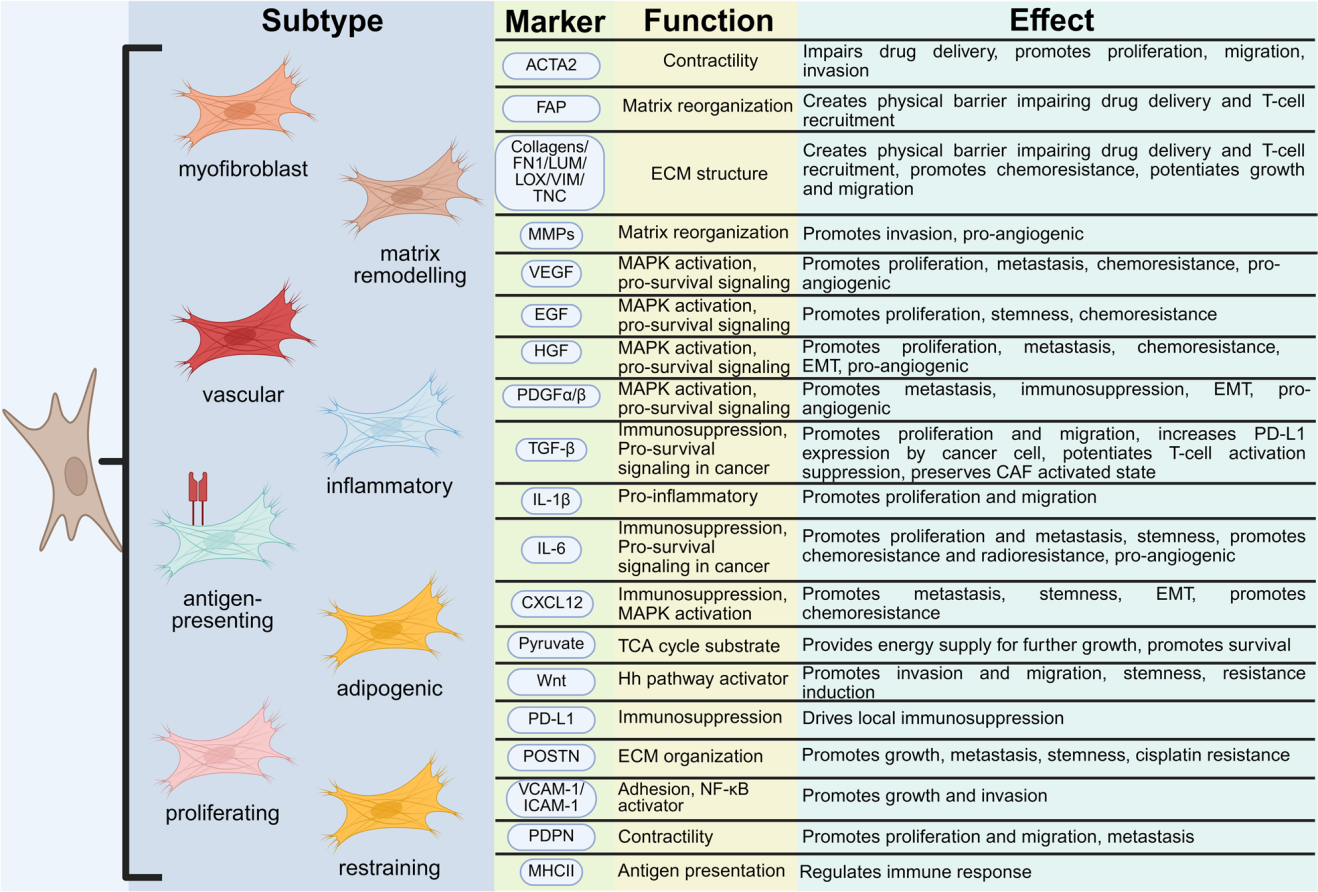
Most commonly described CAF subpopulations and markers. Markers in the figure are not assigned to any specific subtype, as due to the high heterogeneity of CAFs, their expression will differ between cancer types. For example, MyoCAF might have a different expression profile in CRC or LC; or cancer type might have multiple MyoCAFs. Hh – hedgehog pathway. Figure created with BioRender

Biomarkers (Table 1, Fig. 1) play a crucial role in the classification of the various CAF subpopulations, yet they frequently occur in diverse combinations. For example, ACTA2 and FAP can be expressed in two distinct sets of CAFs in head and neck cancer [48], in colorectal cancer [38], or co-expressed in PDAC [42], or breast cancer [34]. Furthermore, CAF classification was not restricted to the presence or absence of specific biomarkers but also considered their expression levels. For instance, subpopulations characterized by high and low expressions of ACTA2 have been identified in PDAC, CRC, breast, and ovarian cancers [5].

### CAF biomarkers as prognostic factors in RCC

Even though several CAF markers are non-specific, multiple studies evaluated their prognostic values based on data from PDAC, colon, lung, breast, esophageal, and prostate cancers [54, 57, 70–79]. Most studies focus on overall survival (OS) or progression-free survival (PFS) using markers such as FAP and ACTA2/αSMA, which are associated with decreased survival rates.

Correlating CAF markers with survival was also performed in ccRCC, where high expression of FAP, COL1A1, COL1A2, COL5A1, COL16A1, EMILIN1, LOXL1, and LUM was correlated with shorter disease-free and overall survival [54, 58, 80, 81]. In the same ccRCC cohort, elevated expression of these markers correlated with advanced stage and higher grade. Similarly, increased CAFs abundance and their increased infiltration were also associated with poorer prognosis. Zhou et al. elucidated the prognostic effect of CAFs in RCC [82]. CAF-scores were based on the expression of prognostic CAF-marker genes. A high CAF-score indicates more activated CAFs/higher expression of selected markers. The study revealed that patients with high CAF scores across ccRCC, pRCC, and chRCC subtypes exhibited poorer clinical outcomes than patients with low CAF scores. The authors also identified 11 and 28 genes related to the survival of patients with ccRCC and pRCC, respectively, whereas no key prognostic genes were identified for chRCC. Based on the identified genes, the authors created CAF signatures that included ADAM metallopeptidase domain 12 (ADAM12) and paired related homeobox 1 (PRRX1) for ccRCC; and cellular communication network factor 4 (CCN4), asporin (ASPN), ACTA2, and TAGLN for pRCC. High-risk patients (showing high expression of signature genes) in both ccRCC and pRCC cohorts experienced poorer prognosis compared to low-risk patients. Moreover, the prognostic value of the CAFs signature showed subtype specificity: the pRCC CC failed to predict prognosis in ccRCC and chRCC, and the ccRCC CAF signature was ineffective in chRCC, yet remained predictive for pRCC patients’ survival. Interestingly, the high-CAF signature of both pRCC and ccRCC patients correlated with decreased response to immunotherapy when compared to groups with low-CAF signatures. It has therefore been suggested that CAFs can have this effect on immunotherapies by promoting T_reg_-mediated immunosuppression. These studies demonstrate that specific biomarker profiles, which vary across tissue or even subtypes of cancer of the same tissue, can be used to predict therapy resistance or drug sensitivity.

**Fig. 2:**
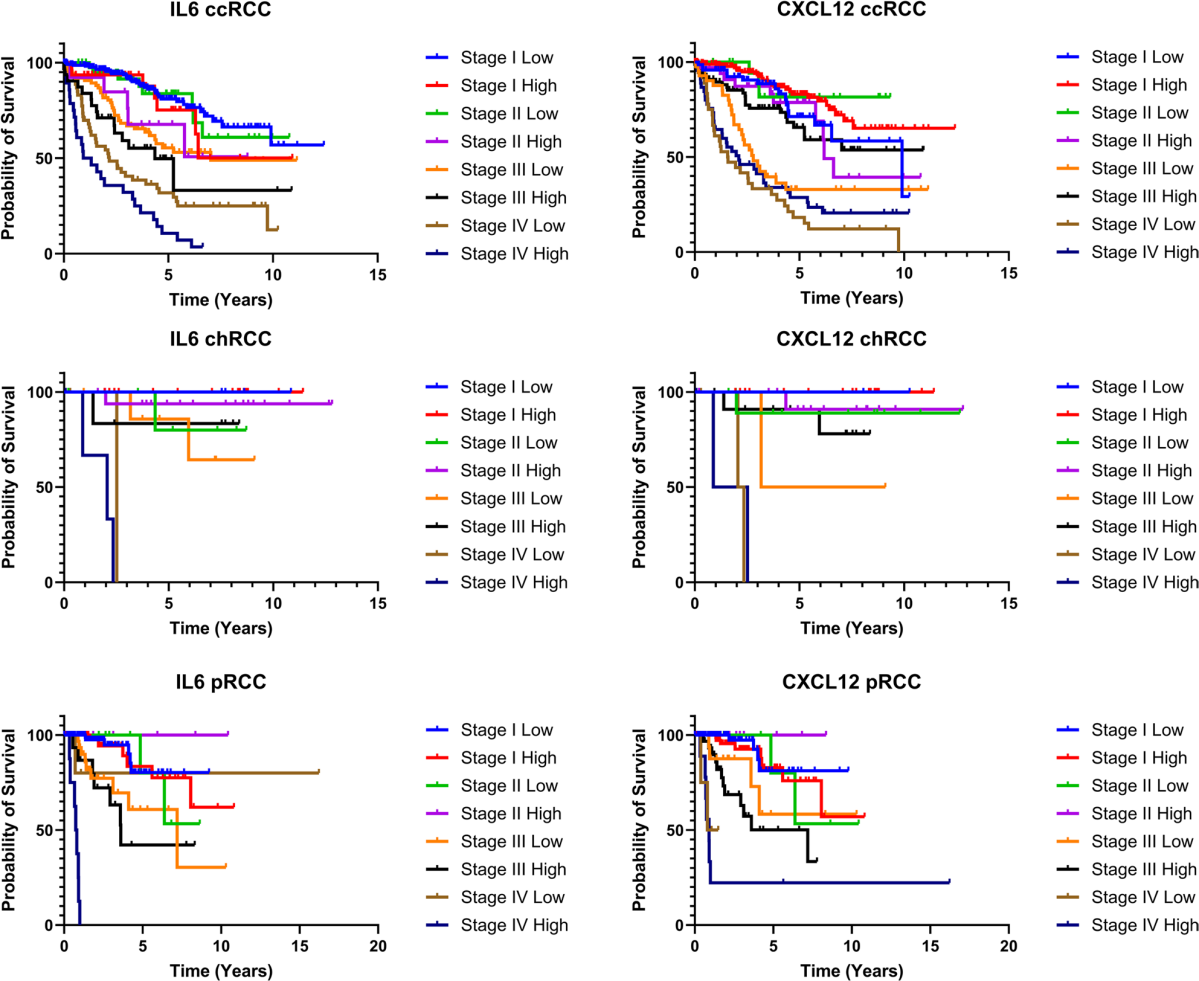
Survival analysis of ccRCC, chRCC, and pRCC based on stage of the tumor and high or low expression of selected markers associated with CAFs. Data sourced from the Human Protein Atlas (www.proteinatlas.org), based on TCGA datasets

While many of the markers proposed by these two research groups can be classified as “CAF-specific”, additional markers related, but not exclusive to CAFs, may still serve as prognostic indicators in RCC. Examples include IL-6 or CXCL12 in ccRCC, both cytokines previously associated with the iCAF phenotype [83]. Their high expression has also been linked with worse prognosis in multiple cancer types. As mentioned above, these cytokines are not exclusively CAF-expressed; however, increased secretion of them by CAFs can still play a crucial contributing factor in survival prognostics. Based on TCGA data (Fig. 2), high expression of IL-6 has been associated with poor survival of stage II and stage IV ccRCC patients, stage IV pRCC, but has not been associated with survival in chRCC. Interestingly, CXCL12 in ccRCC has been observed to have an opposite effect, where high expression of CXCL12 could predict a better outcome for ccRCC with stage III and stage IV tumors. This demonstrates that a high level of these markers can lead to divergent outcomes in tumors of the same tissue. For instance, elevated CXCL12 expression predicts improved survival in ccRCC but is associated with worse outcomes in other cancers [84].

### CAFs’ influence on treatment response

Several studies correlated elevated expression of specific markers with differential treatment responses [25, 72, 79, 85, 86]. For instance, high number of FAP^+^ CAFs in colorectal cancer was correlated with resistance to PD-L1 treatment [25]. A similar response to anti-PD-L1 treatment was observed in PDAC, where a high signature of LRRC15^+^ CAFs was detected [63]. Furthermore, a panel of 12 genes was used to calculate a “risk score” to predict resistance to chemotherapy, including gemcitabine, 5-fluorouracil, and oxaliplatin in PDAC [78]. In this study, “risk score” refers to the probability of reduced survival duration, where a higher score indicates a greater likelihood of disease progression. The risk score was derived from a combination of five markers (RIN2, THBS1, IL-1R1, RAB31, and COL11A1) and analyzed in breast cancer tissue samples [57]. Patients with high-CAF-risk scores exhibited resistance to axitinib, dabrafenib, irinotecan, sorafenib, topotecan, or venetoclax treatment, but sensitivity to alpelisib and epirubicin. Low-CAF-risk patients were sensitive to docetaxel, fulvestrant, lapatinib, ribociclib, and tamoxifen. In lung cancer, a high-CAF-risk signature, involving prognostic genes (EXO1, CCNB1, CLEC3B, and CD302) correlated with poorer anti-PD-L1 therapy response [79]. Interestingly, high stromal Cav-1 expression correlated with improved protein-bound paclitaxel response [85]. Conversely, another study on lung cancer demonstrated that increased expressions of COL1A1, COL1A2, PDGFRB, and ACTA2 were associated with enhanced immunotherapy response [72]. In the same study, COL1A1 and COL1A2 were identified as positive predictors for TKI treatment response.

The CAFs influence tumor progression, immune response, and therapeutic resistance via multiple mechanisms. These include secretion of soluble factors, direct cell-cell contact, ECM remodeling, and metabolic reprogramming. Such interactions have been investigated using various *in vitro* models [6, 7, 9, 10, 18, 87–91].

As described, CAFs directly enhance cancer cell proliferation and promote tumor growth through various mechanisms. They stimulate angiogenesis by secreting factors like SDF1/CXCL12, PDGF, VEGF, and FGF2, which recruit and induce proliferation of endothelial progenitor cells [18, 92, 93]. Moreover, the CAF-driven ECM organization supports the endothelial progenitor cell migration [44]. Clinically, high CAF abundance in lung adenocarcinoma correlated with increased microvessel density [94]. Both of these factors were then associated with worse overall survival of patients when compared to a group containing fewer CAFs. *In vivo* studies further support the pro-angiogenic role of CAFs. For example, in a breast cancer xenograft model, CAFs stimulated endothelial cell growth and recruited endothelial progenitor cells into tumors via SDF-1 and/or VEGF-alpha secretion [91]. This resulted in a significant increase in angiogenesis in the tumor. A subtype of CAFs in breast cancer was named vascular CAF (vCAF), and they expressed genes responsible for angiogenesis and vascular development [16].

### Therapeutic strategies against CAFs and CAFs-induced resistance

**Fig. 3:**
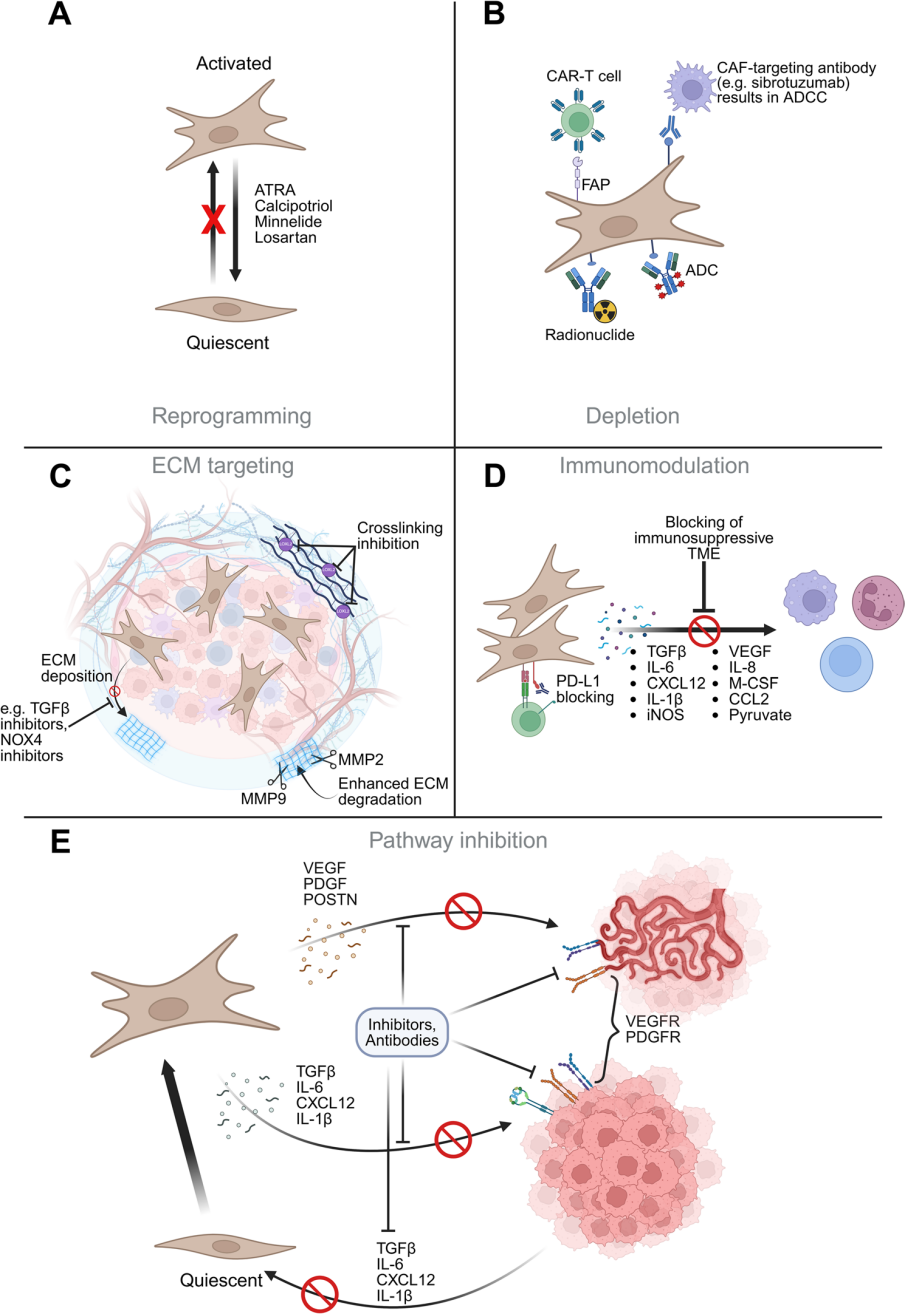
Summary of anti-CAF therapeutic strategies. (**A**) CAF reprogramming to quiescent state; (**B**) Direct CAF targeting; (**C**) normalization of the ECM; (**D**-**E**) targeting of CAF-derived secreted factors. ADC – Antibody-Drug Conjugate, ADCC - Antibody-Dependent Cellular Cytotoxicity. Figure created with BioRender

The implication of CAFs in tumor progression mechanisms makes CAFs an interesting target for novel antineoplastic agents. Furthermore, CAFs are genetically more stable than cancer cells, which could mean that they are less prone to developing treatment resistance [95]. Therefore, various therapeutic strategies targeting CAF molecular markers and CAF-related pathways have been proposed. These strategies, elegantly summarized by others include: (i) CAF reprogramming to quiescent or tumor restraining subtype, (ii) targeting of CAF-derived secreted factors (such as TGF-β, PDGF, IL-6 or CXCL12), (iii) normalization of the ECM, and (iv) direct CAF targeting (e.g., CAF depletion with the help of CAR-T cells) (Fig. 3) [7, 37, 40, 44, 46, 86, 96, 97].

CAFs’ normalization, the process that aims to reverse the CAFs to a quiescent state, can potentially be achieved using various pharmacological agents, such as calcipotriol (vitamin D analog), all-trans-retinoic acid (ATRA), minnelide, or losartan [5, 69, 83, 98]. The use of CAF-normalizing agents has demonstrated promising therapeutic outcomes [99–102]. Fibroblast activation has previously been linked to vitamin A deficiency; therefore, the use of ATRA may lead to reversion of their state [44]. Losartan, an angiotensin receptor II antagonist, reduces TGF-β-mediated activation of αSMA^+^ CAFs, decreasing desmoplasia to improve drug delivery and immunotherapy efficacy [83]. 

Alternatively, targeting CAFs via PDGF ligand traps inhibits tumor progression [103]. These engineered decoy proteins have a higher affinity to the target than antibodies, providing potent pathway blockage [104]. The importance of PDGF signaling can be further supported by a study in ovarian cancer, which demonstrated that PDGF derived from HIF-1α^+^ (hypoxia-inducible factor-1) cancer cells was responsible for activating CAFs [105]. HIF-1α is an oxygen sensor of the cell and acts as a transcription factor, leading to the hypoxia response pathway [106]. HIFs regulate pathways affecting cell metabolism, growth, survival, apoptosis, and angiogenesis. In human head and neck squamous cell carcinomas, hypoxia promotes myCAFs inactivation [107]. This may represent a phenotypic switch rather than full deactivation, *as in vitro* [108] and *in vivo* [109, 110] studies indicate hypoxia can promote the inflammatory phenotype, a finding supported by our own research (unpublished data).

Notably, targeted interventions against specific CAF subpopulations (e.g., chimeric antigen receptor (CAR)-T cells aimed at FAP^+^ CAFs) or blockage of selected CAF pathways may lead to the promotion of subpopulations, such as iCAFs [13]. In the PDAC murine model, inhibition of the Hedgehog pathway led to impaired myCAF function, while expanding the iCAF subpopulation, ultimately establishing an immunosuppressive TME [111]. Another study in murine PDAC has shown that depletion of myCAF populations results in poorly differentiated tumors with much lower OS [98].

TGF-β-mediated suppression of CD8^+^ T-cell activation [112] was clinically relevant in metastatic urothelial cancer; fibroblast-specific TGF-β predicted poor response to anti-PD-L1 treatment (atezolizumab) [113]. This ultimately resulted in the regression of the tumor. Another study performed in PDAC resulted in global blockage of TGF-β signaling led to increased PD-L1 expression by tumor cells [114]. Combining TGF-β inhibitors with anti-PD-L1 treatment improves OS, demonstrating a synergistic anti-tumor effect. Similarly, in a murine PDAC model, cotreatment of Mozobil (Plerixafor, AMD3100), targeting the CXCL12-CXCR4 axis, with anti-PD-L1 reversed FAP^+^ CAF-mediated immunosuppression, enhancing treatment efficacy [115]. Due to the existence of tumor-restraining CAFs, nonspecific pan-CAF treatments risk-averse outcomes, potentially explaining poor efficacy or decreased survival [12]. Most *in vivo* (as well as *in vitro)* research focuses on pancreatic, colorectal, breast, or lung cancer. However, with growing evidence of CAFs’ role in modulating treatment response and the increasing number of TME-targeting clinical trials, it is essential to study CAFs in previously unexplored tumor types, as beneficial CAF phenotypes may vary from one tumor type to another.

Overall, while CAFs represent an interesting therapeutic target, the majority of clinical trials of drugs targeting CAFs fail to give convincing results. This might be attributed to multiple factors, particularly the lack of subpopulation specificity and insufficient CAF-related data among tumor types. Although targeting tumor-promoting CAFs can inhibit tumor growth, monotherapy approaches often offer limited efficacy, advocating for their use in drug combinations. Despite previous failures, several novel anti-CAF therapies are currently undergoing clinical trials [40, 44, 46, 86], highlighting the high expectations and clinical benefits of anti-CAF strategies.

### Targeting CAFs in RCC

Currently, only a few clinically approved drugs specifically target CAFs, and even fewer have demonstrated therapeutic efficacy in ccRCC treatment. Sorafenib presents activity against the MAP kinase pathway, as well as multiple receptor tyrosine kinases, including PDGFRβ [116, 117]. Sorafenib has demonstrated modest efficacy in ccRCC; however, it is no longer considered in current RCC treatment guidelines [117, 118]. Similarly, regorafenib is a multi-tyrosine kinase inhibitor with a wide spectrum of activity against VEGFR1-3, but also PDGFRα [119]. Although a phase II trial demonstrated some activity in RCC, the treatment was toxic and therefore has not been further developed [120]. A small phase II trial, with the use of imatinib, a PDGFR inhibitor, did not demonstrate meaningful clinical activity [121]. Other drugs that could target CAFs, such as olaratumab (mAb-PDGFRα), or vismodegib, a Hedgehog inhibitor, have not yet been tested in this context [122, 123].

Several inhibitors of the fibroblast growth factor receptor (FGFR) family are clinically available, and more are under development. The current indications for FGFR inhibitors are limited to tumors with fusions or mutations of the FGFR1-3 receptors, typically found in urothelial carcinoma or cholangiocarcinoma [124]. The clinical relevance of these agents in tumors lacking FGFR alterations remains unclear, particularly regarding CAF-mediated mechanisms. Dovitinib was tested in patients diagnosed with RCC in a phase II trial and demonstrated minimal activity, with a median PFS of 2.7 months [125]. Similarly, CXCR4 is a potential target for both tumor cells and CAFs. Although Mozobil^®^, a CXCL12-CXCR4 axis inhibitor that is currently approved for the mobilization of hematopoietic stem cells, appears to have promising pre-clinical activity by inhibiting tumor sphere formation [126], data with other CXCR4 inhibitors are less encouraging. LY2510924 was tested in combination with sunitinib in patients diagnosed with metastatic RCC and failed to demonstrate a credible improvement in PFS or OS [127].

New CAF-modulating compounds are currently under development. Several FAP-targeted radionuclides have entered an early phase of clinical trials. Notably, a recent phase II clinical study evaluating ^177^Lu-LNC1004 in multiple solid tumors, including RCC, showed a promising rate of disease control (46%, 13/28) with modest hematotoxicity [128]. Simlukafusp alfa, an immunocytokine binding both FAP and IL-2v, was also tested in several solid tumors in a phase II trial [129], but there is no experience specifically related to renal cancer.

Aforementioned studies were summarized in Table 2.

**Table 2:** Summary of clinical trials that target CAFs in RCC

Clinical study	Drug	Target	Trial Phase	Outcome	REF
-	Mozobil^®^	CXCL12-CXCR4 axis inhibitor	Pre-clinical	Inhibiting tumor sphere formation	[126]
NCT02627274	simlukafusp alfa	FAP-IL2v	I	Manageable safety profile; initial signs of antitumor activity in advanced/metastatic solid tumors; Outcome not specified to kidney	[129]
NCT05963386	^177^Lu-LNC1004	FAP-targeted radionuclide	II	Increased rate of disease control; improved PFS and OS; modest hematotoxicity	[128]
NCT00664326	regorafenib	VEGFR1-3, PDGFRα	II	Activity presented however no further development due to toxicity	[120]
-	imatinib	PDGFR inhibitor	II	No meaningful activity	[121]
NCT00715182	dovitinib	FGFR inhibitor	II	Minimal activity; increased PFS	[125]
NCT01391130	LY2510924 (+ sunitinib)	CXCR4	II	No improvement in PFS/OS	[127]
NCT00073307	sorafenib	RAF, VEGFR-2, VEGFR-3 PDGFRβ, c-Kit	III	Prolonged PFS in previous non-responders	[116, 117]

### CAFs’ isolation strategies

To better understand the cancer-specific effects of CAFs and develop new treatment regimens targeting them, the establishment of biologically relevant *in vitro* models represents an important step. So far, only a limited number of methods for obtaining and maintaining CAFs *in vitro* have been described. These approaches include either isolating CAFs directly from tumors, or activating normal fibroblasts, or other cell types, through exposure to various factors or cellular interactions. The following section describes both approaches.

#### Isolation of CAFs directly from tumors

CAFs have been previously isolated from various human tumors, such as breast cancer [91, 130–133], melanoma [134], head and neck cancer [48, 135], gastric cancer [136], colorectal cancer [60, 137, 138], lung cancer [139], pancreatic cancer [131, 140], liver cancer [141, 142], and ovarian cancer [87, 143]. Generally, the isolation process is based on mechanical, enzymatic, and chemical dissociation or their combinations. In comparison to other cell types, fibroblasts were shown to require stronger dissociation techniques to be extracted from tissues. Waise et al. compared various enzymatic solutions and disaggregation times to isolate fibroblasts from lung tissues [139]. The authors achieved a higher fraction of fibroblasts while using a higher protease-strength enzyme (collagenase P) for a longer duration when compared to lower protease-strength enzymes (liberase DL, TL, and TM).

However, various studies showed that once isolated, fibroblasts change their phenotype *in vitro* [48, 134, 139]. Tirosh et al. identified the unique expression profiles of freshly isolated CAFs and *in vitro* cultured CAFs [134]. The authors found that certain genes, such as complement factors (C1S, C3, C4A), correlated with T cell infiltration in melanoma, however, these genes were expressed solely in freshly isolated CAFs, and not in cultured CAFs. Another study of HNSCC identified that cultured CAFs have lost expression of typical markers and ligands (such as IL6, HGF, TGFB3, CXCL12), failing to induce p-EMT response in cancer cells [48]. It was, however, highlighted by Waise et al. that fibroblasts’ phenotypes shift was due to mechanical changes to tissue culture substrates [139]. Moreover, authors found that expression profiles of CAFs from lung cancer and fibroblasts from non-cancerous tissue were altered already at first passage after culturing either on plastic culture plates or in a three-dimensional (3D) setting with Matrigel^®^. Notably, fibroblasts cultured in a 2D setting showed higher expression of myofibroblast phenotype markers (such as COL1A1 and COL3A1) than fibroblasts cultured in 3D, which showed an increased expression of iCAF markers. However, it is suspected that using more specific media in 3D culture conditions may allow a more accurate phenotype preservation [27]. *In vitro*, no significant difference was observed in selected gene expression between fibroblasts isolated from normal lung tissue and those isolated from lung tumors.

As highlighted above, CAFs were reported to undergo phenotypic changes once they have been isolated and cultured *in vitro*, potentially losing their heterogeneity [48, 134, 139]. However, Orimo et al. showed that isolated CAFs preserve their activated state through multiple passages (up to 10 doublings) without requiring direct interaction with cancer cells or the addition of stimulating compounds in the culture medium [91]. One possible explanation is that upon isolation and subsequent *in vitro* culture, CAFs can transition to a myofibroblastic phenotype in 2D culture conditions, and further preserve this altered, activated, tumor-promoting state without additional stimuli. The authors also observed a positive correlation between the high activation state of specific CAF subtypes and their tumor-enhancing capabilities.

#### Co-cultured fibroblasts activated *in vitro*

An alternative approach to generating the CAFs *in vitro* involves activating normal fibroblasts or other cell types via (trans)differentiation [42, 103, 144]. Various methods have been employed to induce this phenotypic shift, including the use of conditioned media [136, 145–148], hypoxic culture conditions [108–110], exposure to drugs [54], stimulation with cytokines and growth factors [149–151], or co-culture with cancer cells [42, 130, 148]. Further optimization of culture conditions and the incorporation of additional substrates or extracellular matrices may enhance the resemblance to an *in vivo-*like phenotype [27, 152]. Apart from the known activating stimuli, it is possible that the unique state of CAFs can be partially due to some mutations. In that case, CRISPR gene editing could be a useful tool to precisely control the state of the fibroblasts [153]. In this study, the authors modified the promoter of COL1A1 gene, leading to its reduced expression. Matrix derived from modified fibroblasts was then used to assess the effect on the growth of MCF-7 cancer cells. Authors have shown that said matrix did not support cancer cell growth, indicating that the gene editing tools could be utilized to reverse the cancer-promoting effect of CAFs or potentially to induce specific phenotypes of CAFs found *in vivo* that are underrepresented *in vitro* studies.

### *In vitro* co-culture models

**Fig. 4:**
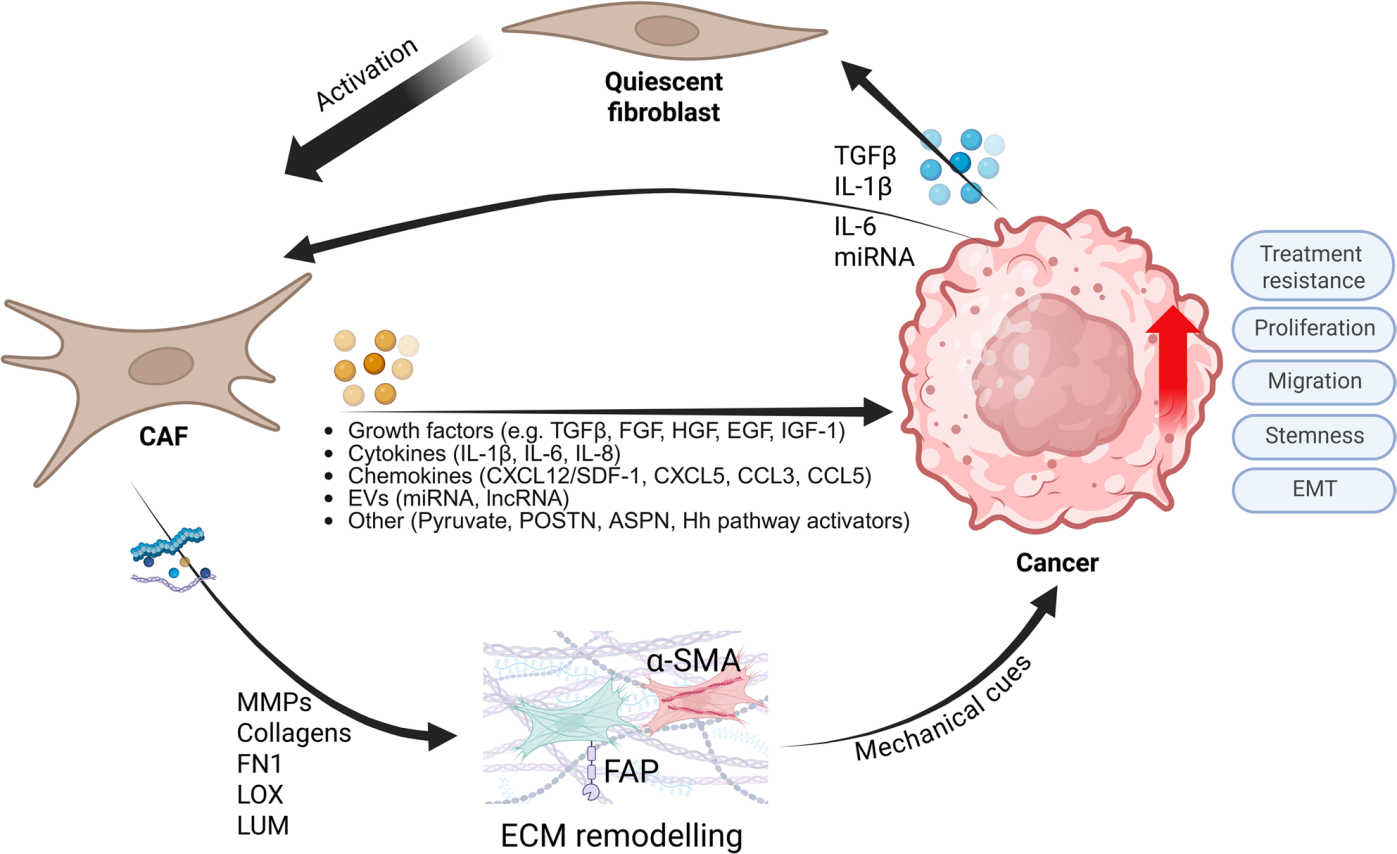
CAF-Tumor crosstalk. Crosstalk takes place either through the exchange of various signaling molecules with cancer cells, meant to either directly bind to cells’ receptors or through modifications of the ECM resulting in mechanotransduction. Figure created with BioRender

CAFs have been used in various co-culture systems, including either direct physical contact with tumor cells or through paracrine interactions. CAF-Tumor crosstalk was depicted in Fig. 4.

**Fig. 5:**
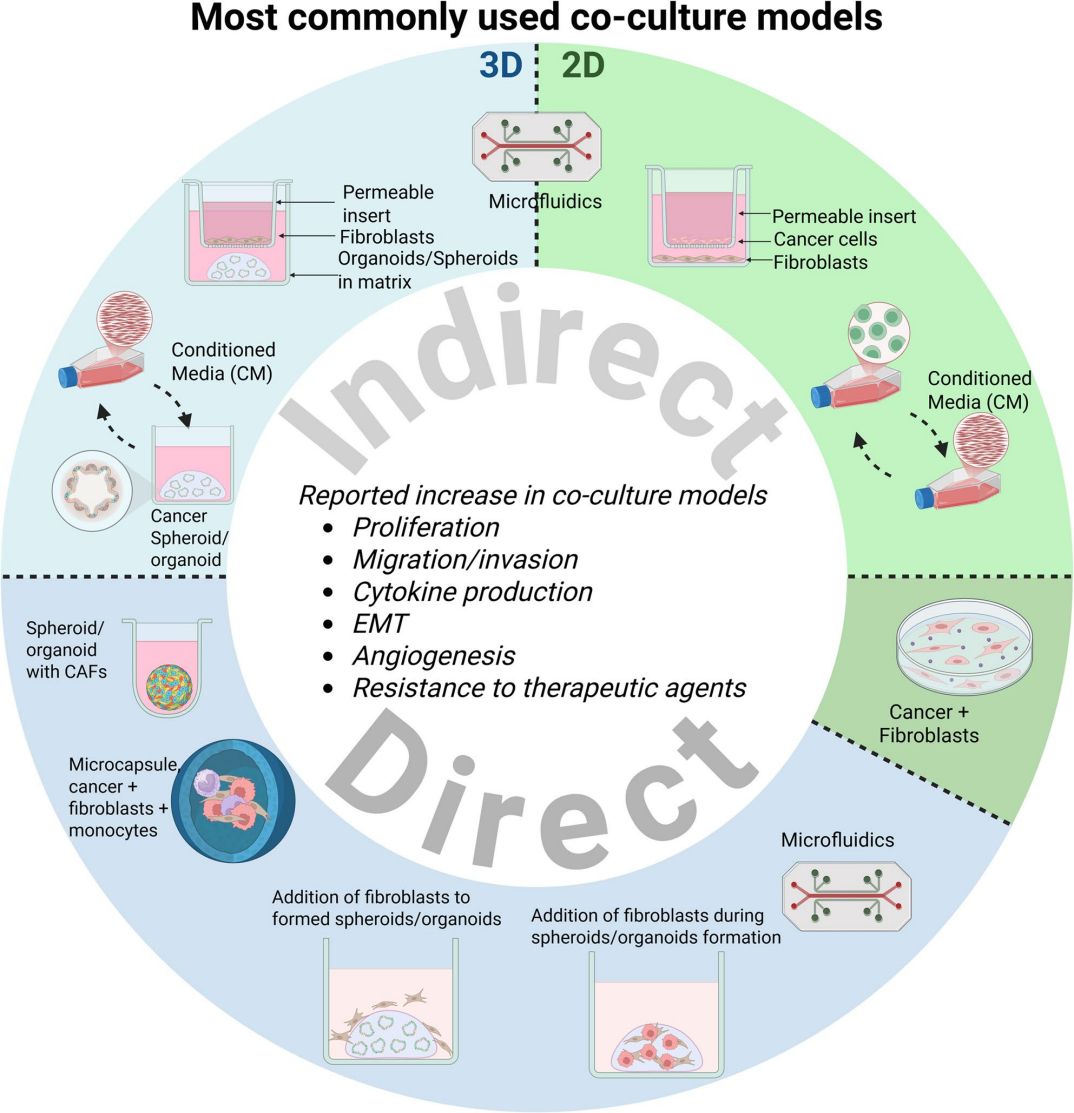
Summary of the most commonly used co-culture models in 2D and in 3D. Figure created with BioRender

These approaches have been applied to 2D or 3D co-culture systems, yielding variable outcomes. This section describes the models for the inclusion of fibroblasts in co-culture and their effect on tumors. We distinguish a direct co-culture as the one allowing juxtacrine and paracrine interactions, and an indirect co-culture model using conditioned media or only allowing paracrine interactions. In this review, we focused on summarizing existing co-culture systems, both two- and three-dimensional. Most used co-culture models have been depicted in Fig. 5

#### Direct 2D co-culture of CAFs

While less commonly reported than other techniques, direct 2D co-culture of CAFs with cancer cells remains a fundamental approach for studying CAF-cancer cell interactions, with documented application in breast [130, 154] and pancreatic cancers [103]. Gao et al. [130] investigated interactions between cancer cells and three distinct fibroblast subpopulations originating from different zones of breast tissue : (i) “normal” zone (distant from the tumor), (ii) the “interface” zone (tumor margin), and (iii) “tumor” zone (core). This spatial classification reveals heterogeneity of fibroblasts‘ function, based on their microenvironment. The interface fibroblasts (INF) or CAFs isolated from the “tumor” zone were shown to enhance cancer cell migratory capabilities and EMT induction, whereas NF induced either a lack or negligible effects under identical conditions. Notably, INFs induced stronger tumor-promoting effects compared to both CAFs and NFs. This enhanced activity was attributed to higher FAP expression and increased secretion of factors, including MMP2, along with factors associated with the MAPK activation pathway in cancer cells. Furthermore, primary CAFs isolated from non-small cell lung cancer (NSCLC) patients induced EMT in co-culture with cancer cells [155]. The requirement for direct cell contact (juxtacrine interactions) in EMT induction was demonstrated by the absence of mesenchymal transition in indirect co-culture.

Enhanced proliferative capacity of breast cancer cells upon direct co-culture with CXCL12-producing fibroblasts was additionally noted by Plaster et al. [154]. In contrast, this increase was much lower in the absence of CXCL12 production. Additionally, the authors noted morphological changes in the cancer cells upon co-culture. Co-culture with CXCL12^+^ fibroblasts caused scattering and spreading of cancer cells, which was consistent with established CXCL12 downstream signaling effect on tumor cell migration and invasion. Similarly, proliferative effects on breast cancer cells were observed as a result of fibroblast IL-1β production [156]. However, authors also noted that this change in proliferation did not require direct cell-cell interactions, as they were also observed in an indirect system using conditioned media. Additionally, co-culture systems can induce specific fibroblast phenotypes [103]. For example, by co-culturing CAFs with PDAC cells, the fibroblasts were reprogrammed into stiffness-induced CAFs (siCAF), a subtype known to suppress CD8^+^ T-cells response both *in vitro* and *in vivo*. This phenotypic shift resulted in an increased PDGFRα-CAF population, confirming the enrichment in siCAFs.

While phenotypic modulation exists between CAFs in co-culture with cancer cells, similar interactions between CAFs and other stromal cells. For instance, primary CAFs isolated from ovarian tumors, when co-cultured with endothelial cells, demonstrated pro-angiogenic features and cord formation promotion [87, 88].

#### Indirect 2D co-culture of CAFs

In CAF-related research, the indirect co-culture approach employing conditioned media (CM) has emerged as the predominant experimental approach [60, 95, 130, 145, 148, 156–159]. CM was derived from CAFs or by co-culturing CAFs with cancer cells on transwell inserts [60, 89, 144, 148, 157, 159, 160]. The letter enabled the exchange of secreted factors but prevented direct cell-cell interactions. These methods have been employed in studies of breast [89, 130, 156, 158], kidney [54], head and neck [135, 159], CRC [60, 157], ovarian [145], esophageal [144], melanoma [148], prostate [95], pancreatic [89], and lung [161] cancers.

Both direct and indirect co-culture models were systematically investigated for fibroblast-mediated effects on CRC cell proliferation and drug response [157]. Their models were either co-cultured with fibroblast cell lines derived from a lung, skin, or colon on a transwell insert, or cultured with the CM of fibroblasts. Using the transwell assay, they showed that colon and skin fibroblasts had very little influence on the proliferation of the SW480, Caco-2, HT-29, and DLD-1 CRC cells, whereas lung fibroblasts had a significantly increased proliferation rate. However, the CM from the same fibroblasts led to different results. While the CM derived from the lung fibroblasts increased the proliferation of SW480 cells, CM from skin and colon fibroblasts led to decreased cell proliferation. This supports our previous hypothesis that fibroblasts of different origins have various effects in co-culture models, and that pan-CAF approaches might lead to unexpected results.

While this study reported differential effects based on CAF’s origin, most studies demonstrated that CAF co-culture generally enhances cancer cell proliferation [135, 157, 159, 160]. Another key hallmark of cancer influenced by CAFs is the enhancement of migratory and invasive capabilities of cancer cells [54]. This effect has been experimentally observed *in vitro* using ccRCC cells (786-O) in a modified Boyden chamber. The model involved seeding 786-O cells, the upper side of an 8 μm filter, and the chamber was then placed on top of a 24-well plate containing a monolayer of confluent fibroblasts [54]. The assay showed that the presence of CAFs resulted in an accelerated migration of cancer cells. Similar findings using another ccRCC cell line (A498) further confirmed the increased proliferative and migratory capabilities of cancer cells upon co-culture with CAFs, but not with normal fibroblasts [162]. This change was associated with high secretion of kynurenine (Kyn), which resulted in increased expression of aromatic hydrocarbon receptor (AhR), ultimately resulting in activation of Akt and STAT3 signaling pathways. Pharmacological inhibition of key signaling pathways targeting AhR (PDM2), AKT (capivasertib), and STAT3 (S1-109) significantly reduced cancer cell proliferation, while both AhR and STAT3 inhibition impaired migratory capacity, while AKT inhibition showed no such effect. A similar approach was used in a study of lung cancer, where either CAFs or normal fibroblasts were co-cultured with cancer stem cells (CSCs) [161]. The authors concluded that CAFs, but not normal fibroblasts, enhanced the invasive capabilities of cancer cells. That effect was later attenuated by the use of a TGF-β inhibitor.

Together, these findings suggest that 2D *in vitro* co-culture models can be of use to study not only the effect of fibroblasts on other cell populations, but also the effect of other cells on CAF phenotype and the global effect on a treatment response. The reviewed findings underscore two key considerations, i.e., (i) the importance of using tissue-matched fibroblasts, and (ii) that both direct and indirect co-culture systems can contribute distinctly to the overall fibroblast-mediated effects.

### 3D co-culture of CAFs

As discussed in the previous section, while 2D culture models can provide valuable data, current scientific consensus favors transition to 3D culture models, as they better recapitulate physiological cell-cell interactions. Therefore, several 3D co-culture models incorporating fibroblasts have been described in recent literature.

#### Direct 3D co-culture of CAFs

CAFs are the most predominant cell component within the TME of pancreatic cancer [163]. For this reason, multiple studies on CAFs performed *in vitro* focus on co-culture with pancreatic cancer cells [89, 90, 109, 164, 165]. Majety et al. [164] studied the role of CAFs in the spheroid models of pancreatic, breast, and lung cancers. Studies revealed that the majority of pancreatic cancer cell lines showed increased cell viability upon co-culture with a lung fibroblast cell line (MRC5). A similar change in cell viability was observed in a minority of tested lung cancer cell lines and half of the breast cancer cell lines. Interestingly, the same effect was observed in co-cultures with primary isolated CAFs. These findings demonstrate that *in vitro* co-culture models can successfully recapitulate the functional roles of tissue-specific CAFs derived from their respective fibroblast populations.

Kuen et al. demonstrated that fibroblast co-culture significantly enhanced spheroid formation capacity and promoted cell survival, whereas monocyte addition did not affect viability [90]. Interestingly, monocytes exhibited strict co-culture dependence for survival, present only in heterotypic spheroids. This triple-population showed that the surviving macrophages differentiated into an immunosuppressive M2-like signature. After further investigation, the authors identified that fibroblasts in the spheroids expressed high levels of IL-6, CCL-2, and IL-8 when co-cultured with cancer cells, and IL-6, CCL-2, and GM-CSF when adding monocytes to the co-culture. Altogether, the M2-like phenotype and the secretion of immunosuppressive cytokines were found to inhibit T-cell proliferation and inhibit activation of CD4^+^ and CD8^+^ T cells. Similar findings were reported in a study in NSCLC [166]. Rebelo et al. demonstrated that alginate microcapsules containing NSCLC cells, CAFs, and monocytes were also capable of recreating an immunosuppressive TME that was enriched in a mixture of cytokines and chemokines (e.g., IL-4, IL-10, IL-13, CCL24, CXCL_1_), ECM proteins (COL1, COL4, FN1), and ECM remodeling factors (MMP1, MMP9). These TME alterations promoted both tumor cell invasion and monocyte differentiation into M2-polarized macrophages characterized by immunosuppressive properties.

The CAF-mediated acceleration of cancer cell proliferation has been consistently demonstrated across the 3D models, including head and neck cancer [135], breast [156], pancreatic cancer [167], or squamous cell carcinoma [161]. The organoid model of estrogen receptor breast cancer cells (ER + BCC) showcased that the presence of normal fibroblasts led to increased proliferation of both fibroblasts and cancer cells [156] as a consequence of high IL-1β expression by fibroblasts, whereas increased proliferation of fibroblasts was due to other factors, such as PDGF-BB produced by cancer cells. The authors observed upregulated expression of IL-6, IL-8, and CCL7 in fibroblasts in co-culture. These cytokines have been previously shown to stimulate the expression of PDGF-BB by cancer cells [168]. Interestingly, the study revealed that normal fibroblasts and CAFs shared similarities in co-cultures, with both populations exhibiting similar factor expression of various factors and similarly affecting the cancer cells. The study challenges the necessity of NFs, demonstrating that distinct fibroblast populations shared common pathways in supporting cancer cell proliferation. This seems to be supported by an *in vivo* study in PDAC, where CAFs were inactivated via ATRA [167]. Although tumor size increased compared to control (tumors without CAFs), the increase was smaller than in CAF-rich conditions. However, in multiple *in vitro* studies, cancer cells transform normal fibroblasts into CAFs. This suggests that the presumed NFs in this study might represent *in vitro-*activated CAFs [156]. While freshly-isolated CAFs initially presented significantly higher expression of IL-1β compared to normal fibroblasts, this differential was lost over time in culture, and CAF-derived IL-1β levels decreased to match those of NFs. Both the CAF and normal fibroblasts presented the iCAF phenotype.

A spheroid model of squamous cell carcinoma (SCC) lung metastasis was grown in co-culture with CAFs or NFs [161]. The presence of either type of fibroblasts resulted in increased spheroid formation, which was more pronounced in the condition containing CAFs. Moreover, this increase was alleviated by the use of a TGF-β inhibitor, but only in the CAF-containing spheroids. It was therefore concluded that (i) TGF-β released by CAFs plays a crucial role in self-renewal of CSCs, and (ii) there are other factors produced by either fibroblasts that contribute to this change. Interestingly, a study in PDAC showed that CAF-cancer cell interaction in some cases might not only be beneficial for cancer cells, but rather be required for their survival, which was fully dependent on CAF-derived factors [165]. The authors identified three PDAC subtypes, two of which were independent from exogenous Wnt ligands and could grow and survive in a culture without the addition of Wnt, and one dependent on exogenous Wnt ligands for growth and survival. PDAC cells progressively developed niche independence during disease progression. It was therefore concluded that the inclusion of CAFs in the organoid co-culture system resulted in supported cancer cell growth and survival, indicating that Wnt ligands expressed close to cancer can promote tumor progression and metastasis. Moreover, this effect was not observed when CAF-conditioned media or an indirect co-culture model were used, demonstrating the importance of juxtacrine interactions. Cancer cell growth promoted by CAFs was then reversed by the use of a porcupine inhibitor that stops the production of Wnt ligands. Use of Wnt pathway inhibitors may be crucial in PDAC development, as high expression of Wnt ligands by more aggressive, independent cancer cells was linked with poor survival and metastatic progression of PDAC.

Apart from proliferation, the secretion of growth factors and cytokines was also shown to be affected [164]. Upon addition of fibroblasts into the culture, a shift in expression of secreted factors was observed in a cancer type-specific manner. Main changes were observed in the expression of EGF, HGF, and IL-6, all of which were previously associated with tumor progression, resistance, and inflammation.

Guo et al. proposed a method to study interactions between cancer, endothelial cells, and CAFs using a 3D direct co-culture “ring assay” [89]. The authors observed enhanced vascularization in a pancreatic cancer tri-cancer model (endothelial: CAF: cancer cell ratio of 1:1:10, respectively), with this ratio demonstrating maximal pro-angiogenic effects. While this model was proposed for investigating cancer stemness modulation, subsequent analysis revealed no significant changes in stemness markers during CAF co-culture [135]. The model proposed by Guo et al. might also be utilized to study EMT, which allows cancer to gain mobility and contributes to increased invasive capabilities and metastatic activity [169]. Such a change was observed in the aforementioned direct spheroid model. EMT was also studied in a CRC organoid [137] and breast cancer spheroid co-culture model [170]. In the CRC organoid model, the CAFs were found to induce partial EMT in specific cancer cell subpopulations [137], effectively recapitulating key features of aggressive mesenchymal-like colon cancer. Additionally, these CAF-educated organoids exhibited elevated expression of immunosuppressive factors, including TGF-β1, VEGFA, and lactate, known to potentially inhibit T-cell proliferation. In the breast cancer model, the effects of CAFs with altered expression of Tiam1 (T-cell lymphoma invasion and metastasis-inducing protein 1) have been studied, which have previously been shown to alter proliferation, invasion, and EMT in cancer [170]. The co-culture with Tiam1-deficient CAFs led to increased cancer cell invasion into the matrix, whereas Tiam1-overexpressing CAFs led to decreased invasion of cancer. Interestingly, breast cancer cells isolated from spheroids with Tiam1-deficient CAFs showed enhanced migratory capabilities, EMT, and CSC characteristics, whereas the opposite effect was observed for cancer cells isolated from the Tiam1-overexpressing CAF co-cultures. Moreover, these changes in cancer cells persisted for weeks after isolation from spheroids, indicating that a short exposure (2 days) to CAFs can have a long-lasting effect.

#### Mechanopathological niche

The mechanopathological niche containing CAF-related stiffness, tumor hypoxia, and extracellular matrix modeling orchestrates tumor progression and therapy resistance.

Tumor stiffness represents an important tumor feature that has been shown to influence multiple hallmarks of cancer, including stem cell differentiation, metastatic potential, and resistance to apoptosis [171]. CAFs isolated from colorectal cancer were divided into 6 distinct populations [138], each exhibiting heterogeneity in matrix remodeling, either stiffening or softening of the ECM. This heterogeneity was, however, lost when patient-isolated CAFs were directly co-cultured with CRC cell lines. Notably, co-culture with the invasive CRC cell line (HCT116) reduced matrix stiffness across all experimental replicates. In contrast, a separate study of CRC organoids reported CAF-dependent matrix stiffening, an effect absent in both organoids or CAF monocultures [137]. A decrease in the stiffness of the matrix was shown to correlate with decreased drug efficacy in an *in vitro* [172] study, whereas an increase in stiffness was linked with increased metastatic properties [173]. A decrease in stiffness, however, is caused by high activity of degradation enzymes such as MMPs, whereas an increase in stiffness is caused by increased matrix deposition and crosslinking. This shows that not only does the direct interaction of CAFs with cancer through direct contact or secretion of soluble factors play a role in tumor progression, but also indirectly by modulation of the entire TME.

Another tumor characteristic, often omitted in *in vitro* investigations, is tumor-related hypoxia. Hypoxia was shown to impact the growth and treatment response of tumor cells, as well as in co-cultures with CAFs [109]. Upon culture in hypoxic conditions, pancreatic stellate cells (PSCs), a precursor of CAFs in pancreatic cancer, showed enhanced expression of molecules associated with inflammatory CAF phenotype (such as IL-6), and a decrease in myCAF markers (such as ACTA2). Co-culture of PSCs with cancer organoids also resulted in elevated IL-6 expression. The incubation of co-culture in hypoxia further elevated the expression of this marker. This fact can be attributed to increased production of IL-1α by cancer in hypoxic conditions - a molecule which has been attributed to the induction of an iCAF phenotype. IL-1α then induces expression of leukemia inhibitory factor (LIF) *via* NFκB in PSCs, which was shown to promote progression of PDAC [30]. Blocking of LIFs’ function by use of LIF-neutralizing antibodies resulted in reduced expression of IL-6, presenting a potential target in blocking CAF-cancer interactions. Hypoxia-associated response was also studied in an organoid co-culture model of ovarian clear cell carcinoma [105]. The authors demonstrated that HIF-1α expression in an ovarian cancer co-culture model was specifically induced by CAFs, rather than by hypoxic conditions alone. This finding highlights the essential role of tumor-stroma interactions in mediating cellular responses to hypoxic conditions. Moreover, complementary studies revealed that the combination of hypoxia and CAFs promoted therapeutic resistance *in vitr*o.

The physical role of CAFs was investigated in a breast cancer brain metastasis model [174]. Two novel 3D culture systems were established using (i) a perpendicular slide chamber and (ii) applying a 3D-embedded culture method to reflect brain metastasis conditions. These direct co-culture models allowed recapitulation of the influence of CAFs on (i) disruption of the blood-brain barrier and (ii) invasive migration of cancer cells when interaction with CAFs was present. The results confirmed that these 3D models reliably recapitulated the initial steps of blood-brain barrier transmigration by cancer cells via enhanced vascular permeability, micro-metastasis, and colonization.

The next frontier in the development of physiology-related models lies in developing models that are not only three-dimensional but also patient-specific stroma cells. The incorporation of patient-derived CAFs into 3D co-culture models represents a pivotal advancement in this direction, offering a powerful tool for precision oncology. The use of immortalized CAF lines, while convenient, in reality fails to capture the critical heterogeneity often present in tumors [175].

By isolating CAFs directly from patient tumor samples (either surgically resected or from biopsies), researchers can preserve this unique, patient-specific CAF repertoire, including its associated extracellular matrix (ECM) composition and signaling networks. Patient-derived CAF-based 3D models serve as a “patient avatar” for ex vivo therapeutic testing. These models can more accurately predict a patient’s response to both conventional and targeted therapies. For instance, they can reveal stroma-induced resistance mechanisms, such as how specific CAF subtypes create a physical barrier to drug penetration or secrete survival factors that protect tumor cells from chemotherapy or targeted agents [86, 176, 177].

As TME plays an important role in tumor progression, the inclusion of its elements in co-culture should be considered. Organoid co-culture systems can be difficult for new users and expensive due to the use of various factors in culture media. A solution was proposed in a CRC model, which required only a simple culture medium without the growth factors addition [151]. This protocol provides a robust way of co-culturing patient-derived CRC organoids with TGF-β pre-activated fibroblasts (CCD18co). This model can also incorporate primary CAFs. While our lab recently developed a patient-derived organoid protocol, here we focused on normal kidney and RCC tissue [178]. The protocol by Wallisch et al. relies on fibroblast migration from 2D onto the 3D structures placed atop the stromal compartment. This approach can result in heterogeneous organoid populations within the same culture, where some can get encapsulated by fibroblasts, whereas others can only partially interact with fibroblasts. In contrast, our laboratory protocol introduces a method to generate single organoids, which can then be mixed with the stromal compartment. This ensures a homogenous organoid population across the samples and improves robustness and reproducibility.

Furthermore, the co-culture models containing patient-derived CAFs are essential for testing the efficacy of drugs that aim at disrupting CAF-tumor crosstalk, i.e., FAK inhibitors, Hedgehog pathway inhibitors, or anti-fibrotic agents [179]. These models can identify patients likely to benefit from a specific combination. They offer a rationale for personalized regimens that go beyond genetic data by testing without a patient’s unique tumor microenvironment. Key challenges in the generation of these models include the need for consistent isolation of both cell types, maintaining the *in vivo* phenotype of CAFs in culture, and scaling the technology for high-throughput drug screening [83, 180].

#### Indirect 3D co-culture of CAFs

Although used less often than 3D direct co-cultures, the interplay of CAFs and cancer cell-secreted factors exists without direct cell-to-cell contact. The squamous cell carcinoma CSC spheroid formation upon co-culture with CAFs and NFs was enhanced in the presence of CM from the CAFs [161]. This effect was attenuated with TGF-β inhibitor (LY2157299), but no change was observed in the conditions containing conditioned medium from NFs. This suggested that TGF-β1, which was highly concentrated in CAF CM, but not in NFs CM, was responsible for this change.

Koh et al. used a 3D indirect co-culture model, using either CM or transwell inserts, to assess the impact of fibroblasts from different origins on the proliferation and apoptosis of CRC cell lines [157]. Similarly to 2D settings, the transwell model in 3D increased the proliferation of SW480 cells in co-culture with lung fibroblasts but not with skin or colon fibroblasts. Colony formation was also affected. As in 2D, the use of CM resulted in an increased proliferation of cancer cells in contact with lung fibroblasts CM, but decreased proliferation in the presence of skin and colon fibroblasts. None of the co-culture methods affected the apoptotic rate of the cancer cells, but the 3D models were characterized by a higher apoptotic rate compared to the 2D model. A similar effect on the proliferation of cancer cells was also shown by Chatterjee et al. in a breast cancer model [156]. Authors showed not only increased proliferation of cancer cells but also fibroblasts in a 3D direct co-culture. To assess if this change was dependent on juxtacrine or paracrine interactions, the authors utilized CM from fibroblasts, cancer cells, and co-cultured organoids. The authors observed that in both fibroblasts and cancer cells, only the CM derived from the co-culture model significantly increased cell proliferation. This suggests once again that the interaction of the fibroblasts with cancer is needed for the secretion of pro-proliferative factors.

In a CRC spheroid co-culture model, similarly to direct 3D organoid co-culture, an increased EMT signature, as well as increased migratory capabilities, were noted [181]. This model thus recapitulated the EMT-like state of invasive properties of early metastatic tumors. The transition was further confirmed by observation of altered localization of β-catenin, increased expression of mesenchymal markers, and decreased expression of epithelial markers. In the CRC model, both indirect and direct co-cultures led to similar conclusions, showcasing a crucial role of CAFs in CRC progression and metastasis.

The indirect organoid model of PDAC with CAFs was utilized to study hypoxia effects [110]. Initially, the authors found that co-cultures containing pancreatic stellate cells and pancreatic cancer cells in a transwell model resulted in the acquisition of an inflammatory CAF phenotype. Expression of inflammatory molecules such as IL-6, CXCL1, and LIF was further elevated in the co-cultures in hypoxic conditions (1% O_2_). This induction was absent in hypoxic monocultures, although hypoxia itself triggered IL-1α expression in cancer cells. Neutralizing IL-1α prevented iCAFs formation. Moreover, co-culture under hypoxia further increased IL-1α, demonstrating bi-directional interactions. The same observations replicated in a direct co-culture model highlight that hypoxia-driven paracrine interactions without direct cell-to-cell contact are essential for modulating cancer cells and CAFs.

Two limitations of the indirect co-cultures were highlighted by Jeong et al. [182]. Transwell inserts create a non-physiological distance between the cell compartments and often contain relatively large amounts of medium, diluting secreted factors, limiting their ability to exert any effect. Use of conditioned media, however, lacks the bilateral cell-cell interactions. To address those limitations, the authors developed a microfluidic chip-based method that more accurately mimics paracrine cancer-CAF interactions in a physiologically relevant setting. They observed changes in both compartments: induction of a CAF state and an increase in their migratory capabilities, followed by increased growth rate of the CRC spheroids. Surprisingly, within the same spheroid, the Ki67 expression, a proliferative marker, was significantly reduced. Inhibited proliferation of cancer cells was previously linked with EMT [92]. EMT seems to be further supported by increased expression of fibronectin, a stromal marker. This model enables the study of paracrine-stroma interactions while overcoming the two major limitations of indirect co-culture models.

A recent advancement in the field involves the move away from standard basement membrane extracts (such as Matrigel^®^) toward engineered, biomimetic scaffolds to culture patient-derived organoids (PDOs) and CAFs. These synthetic hydrogels are designed to better replicate the biochemical and biophysical properties of the native tumor stroma. For instance, hyaluronan–gelatin-based hydrogels have emerged as a key platform, given that hyaluronic acid is a major component of the desmoplastic stroma in many cancers, such as pancreatic [183, 184] or breast cancer [185]. The adoption of these defined systems addresses several limitations of animal-derived matrices. By tuning the stiffness, ligand presentation, and proteolytic degradability of the hydrogel, researchers can create a microenvironment that more faithfully supports the complex crosstalk between tumor cells and CAFs [186]. The study of Luo et al. has demonstrated that PDOs co-cultured with autologous CAFs in these biomimetic hydrogels exhibit more *in vivo*-like growth patterns [187]. These PDO-CAF co-cultures proved to be effective platforms for testing standard-of-care therapeutics, positioning them as a valuable tool for advancing personalized cancer treatment.

### CAFs affect drug response *in vitro*

As described in previous sections, CAFs modulate therapeutic response *in vivo*. These observations have been consistently recapitulated *in vitro* in co-culture systems [54, 95, 105, 135, 159, 160, 162, 164, 166, 167, 182, 188–191], demonstrating the importance of CAF-mediated drug resistance phenotype. We organized the information below according to its organ of origin.

CAFs were shown to alter the response to therapeutic agents. A change in treatment response was observed in a 2D model of breast cancer upon the use of CAF-derived CM [158]. The study found that CAF-derived IL-6, which activates the STAT3 signaling pathway, promotes radioresistance. The use of STAT3 inhibitors or anti-IL-6 antibodies slowed the growth of cancer cells and blocked the CAF-induced radioresistance.

The fibroblast-induced resistance to tamoxifen observed in the 3D breast cancer model was accompanied by hyperphosphorylation of Akt and MAPK [192]. This model also exhibited reduced sensitivity to the MEK1/2 (U0126) and PI3K/Akt inhibitor LY294002. As both pathways play an important role in tumorigenesis and can regulate chemotherapeutic resistance in cancer, their activation provides a plausible mechanism for the observed phenotype [190, 191]. Resistance to anti-EGFR therapy was also shown in a pancreatic cancer direct spheroid model [164]. The authors reported that co-culturing with CAFs led to a reduction in response to cetuximab. In contrast, treatment with R1507 (anti-IGF1R treatment) decreased cancer cell viability.

In a 3D spheroid model of breast cancer, upon co-culture with CAFs, a decreased viability was observed when anti-IL-6 treatment was used [164]. Beyond directly modulating cancer cells’ response to therapeutics, CAFs, through excessive ECM deposition/remodeling, can establish a physical barrier preventing access to the drugs.

In a 2D ccRCC model, Ambrosetti et al. demonstrated this barrier effect, preventing the uptake of sunitinib by tumor cells [54]. Notably, treatment with the TGF-β receptor inhibitor LY2109761 effectively disrupted this barrier. Interestingly, this drug was shown to decrease the viability of CAFs; however, it did not affect the ccRCC cells. Both ccRCC co-culture models [53, 160], the results confirmed an increase in resistance induction to sunitinib; however, two different mechanisms of resistance were proposed. A drug response was also linked to the origin of the fibroblasts used [157]. Apart from the effect of CAFs on chemotherapy resistance, many studies have also reported CAF-induced resistance to targeted therapies. As such, the importance of Akt/STAT3 signaling pathways on said resistance was highlighted in the ccRCC 2D co-culture model [162]. CAFs from RCC were secreting high amounts of Kyn because of upregulated expression of tryptophan-2,3-dioxygenase (TDO), ultimately activating Akt/STAT3 signaling pathways. Furthermore, Kyn produced by CAFs, and as a result Akt/STAT3 pathway, was associated with decreased response of A498 cells to sorafenib and sunitinib. Use of the TDO inhibitor (LM10) suppressed the increased resistance to sunitinib and sorafenib. Similarly, STAT3 inhibition with S1-109 reduced drug resistance, whereas inhibition of Akt by capivasertib failed to restore treatment sensitivity. This suggests that Kyn might mediate its effects through distinct downstream signaling pathways.

A drug response was also linked to the origin of the fibroblasts used [157]. In this CRC model, the presence of lung fibroblasts led to decreased response to 5-FU and doxorubicin, whereas in 3D co-culture, decreased response was observed when treated with irinotecan, camptothecin, and doxorubicin. This effect was not observed when skin or colon fibroblasts were used. In the same study, both 2D indirect and 3D indirect settings, lung fibroblasts led to decreased response to XAV939 (Wnt/β-catenin inhibitor), whereas colon and skin fibroblasts did not affect the treatment. Wnt/β-catenin signaling was shown to be an important pathway in the resistance of the pancreatic cancer 2D model to a chemotherapeutic agent: mitoxantrone [193]. Fibroblast-derived WNT16B was identified as a key mediator of chemotherapy resistance via the Wnt/β-catenin pathway. Chemotherapy-induced genotoxic stress triggered NF-κB-mediated WNT16B upregulation in fibroblasts, and its secretion promoted resistance in cancer cells. In CRC spheroid on the chip models, a potential chemoresistance mechanism was identified [182] where CAF-induced EMT reduced doxorubicin uptake by cancer cells, which was accompanied by elevated cancer-cell-derived fibronectin that potentially decreased the availability of the drug.

2D models and a 3D spheroid model of head and neck cancer, when co-cultured with CAFs or by use of conditioned media, were shown to be partially resistant to cetuximab, an anti-EGFR agent [135, 159].

In a lung cancer model, CAFs have also been linked to therapeutic resistance [160]. The authors found that inhibition of plasminogen activator inhibitor-1 (PAI-1) resulted in increased response to cisplatin, due to suppression of myofibroblastic properties of CAFs and their increased apoptosis.

Resistance to EGFR-targeting drugs was also shown in the lung cancer model [194]. Authors have shown that when co-culturing lung cancer cells with hepatocyte growth factor (HGF)-producing CAFs, cancer cells have gained resistance to EGFR-TKIs. This resistance, however, was overcome by combining anti-HGF antibodies or HGF agonists with gefitinib.

Cisplatin resistance was not observed in a direct co-culture spheroid model of NSCLC containing CAFs and monocytes; however, the same model showcased increased resistance to paclitaxel [166].

2D model of prostate cancer showed that co-culture of cancer cells with either CAFs or NF reduced cell death after treatment with doxorubicin, paclitaxel, and mitomycin C [95]. The same effect was observed upon the use of CM from either of the fibroblasts. The resistance to doxorubicin was attributed partially to CAF-derived glutathione (GSH), which prevents accumulation of the drug in cancer cells, attenuates drug-induced DNA damage and p53 induction, induces ROS production in cancer, ultimately contributing to cancer cell survival. A similar mechanism of resistance was described in ovarian cancer [188]. The authors found that CAF-derived GSH and cysteine were responsible for blocking cisplatin accumulation in ovarian cancer cells. However, CD8^+^ T-cells were found to counteract the CAF-mediated resistance by expressing IFNγ, which restricts cysteine production and promotes GSH degradation. It is, however, possible that more molecules can affect the cisplatin resistance in ovarian cancer. As such, CCL5 was identified to be another possible factor [189]. Cisplatin treatment has been shown to induce CCL5 expression by CAFs, which led to increased phosphorylation of STAT3 and Akt.

Since CAFs can be co-cultured with other components of the TME than cancer cells, Sulaiman et al. demonstrated that the presence of CAFs derived from ovarian cancer samples, in co-culture, protected the endothelial cells from an anti-angiogenic drug Lenvatinib [87]. Therefore, contributes to the development of resistance to anti-angiogenic compounds. While mechanistic studies incorporating cancer cells, CAFs, endothelial, and immune cells may complicate the identification of the specific cellular compartment responsible for observed effects, such pluricellular systems are critical for assessing drug treatment efficacy. Another study in ovarian cancer demonstrated CAF-mediated carboplatin resistance in a spheroid model driven by PDGF signaling [105]. PDGF derived from HIF-1α^+^ cancer cells was responsible for increased activation of CAFs, resulting in activation of pro-survival signals that enhanced chemoresistance. Pharmacological inhibition of PDGF reversed this chemoresistance and restored carboplatin sensitivity.

A 3D direct co-culture of pancreatic cancer organoids with CAFs showcased a decreased response to gemcitabine, presumably by induction of EMT of cancer cells and/or impaired drug delivery through excessive ECM deposition. Interestingly, the addition of collagenase to the co-culture treatment led to a slight increase in treatment efficacy, when compared to treated co-cultures, however, the viability of cancer was still significantly higher than that of the pancreatic cancer organoid themselves.

Resistance in melanoma was also noted [195]. In a direct co-culture model, 7 of used BRAF-mutated melanoma cell lines, cancer showed resistance to a RAF inhibitor, PLX4720. To determine if the resistance was caused by direct contact or secreted factors, the authors used CAF-derived conditioned medium, which had a similar effect. The authors found that CAF-derived HGF was responsible for the resistance. These findings were confirmed *in vivo*, where only 1 out of 34 patients responded to the treatment, and interestingly, the patient was found not to have expression of HGF in the tumor. Thus, HGF in melanoma can be used as a predictive marker.

As highlighted by several of the cited studies (Table 3), combination therapies represent an interesting strategy to combat tumors. In particular, targeting or normalizing CAFs along with anti-tumor agents shows promising results in overcoming therapy resistance. Thus, these combinations should be further exploited in future studies across various cancer types.

**Table 3:** Summary of studies showcasing CAF-induced resistance *in vitro*

Cancer Type	Tumor Model/co-culture type (direct/indirect)	Cell line/patient cells	Inclusion of CAFs caused resistance to	Proposed mechanism of resistance	Resistance overcame by	REF
Breast	2D indirect (conditioned medium)	T47D, MCF-7, MDA-MB-231	radioresistance	CAF-derived IL-6 activated the STAT3 signaling pathway in cancer, resulting in increased resistance	IL-6 neutralizing antibody (MA5-23698); Stattic (STAT3 inhibitor)	[158]
	3D direct, spheroid	EIII8, MCF-7, MDA-MB-231	tamoxifen	Hyperactivation of Akt, independent of EGFR or IGFR signaling	Partially, but not fully by MAPK (U0126) or PI3K/Akt (LY294002) inhibitors	[192]
	3D direct, spheroid	BT20	cetuximab	CAF-derived IL-6 has been previously linked with resistance to various drugs	In the study anti-IL-6 treatment resulted in decreased viability, however no combination was tested if resistance to cetuximab was overcome	[164]
ccRCC	2D indirect/direct ccRCC	A498	sunitinib sorafenib	Enhanced activation of Akt and STAT3 signaling through CAF-derived Kyn which up-regulates expression of AhR	DMF - AhR inhibitor LM10 – TDO inhibitor S1-109 – STAT3 inhibitor	[162]
	2D indirect	786-O	sunitinib	Creating a physical barrier blocking the availability and uptake of the drug	LY2109761 (TGF-β receptor inhibitor) – reversed the barrier and allowed to higher accessibility of sunitinib to cancer	[54]
CRC	2D/3D indirect	SW480, HT-29, DLD-1, Caco-2	5-fluorouracil, doxorubicin, camptothecin, irinotecan, XAV939	-	-	[157]
	3D indirect spheroids on chip	HT-29	doxorubicin, paclitaxel	Induction of EMT which leads to elevated expression of stromal fibronectin by cancer cells, potentially decreasing availability of the drug	-	[182]
Head and neck	2D indirect (conditioned medium and transwell insert)	UT-SCC-9, UT-SCC-24 A, UT-SCC-19 A, UT-SCC-76 A	cetuximab	CAF-derived MMPs contribute to observed resistance	Partially by MMP inhibitor III	[159]
	3D direct spheroids	Patient derived - LK0902, LK0917, LK1108	**Increased effect** cetuximab	CAFs led to increase of EGFR-expression by cancer cells, which is a target of cetuximab	-	[135]
Lung	2D indirect (transwell insert)	A549, PC-9, LLC	cisplatin, afatinib	CAF-derived PAI-1 was reported to induce EMT of cancer and through positive feedback loop, promoting myoCAF properties	SK-216 - PAI-1 inhibitor – suppressing myoCAF	[160]
	2D indirect (conditioned medium and transwell insert)	PC-9, HCC827	gefitinib	CAF-derived HGF restores Akt signaling pathways, conferring resistance	Anti-HGF neutralizing antibody; NK4 – a natural HGF inhibitor	[194]
Melanoma	2D direct/indirect (with use of conditioned media)	A2058, C32, COLO 829, G-361, MALME-3 M, SK-MEL-28, SK-MEL-5	PLX4720	CAF-derived HGF was identified to contribute to treatment resistance through acting on MAPK and Akt pathways	Dual inhibition of RAF and HGF or MET reverses observed resistance	[195]
NSCLC	3D direct Spheroid with addition of monocytes	NCI-H157	paclitaxel, no effect on cisplatin or BLZ945 (CSF-1R inhibitor)	Protection of cancer from apoptosis	Although based on ATP levels resistance was not overcome, treatment with cisplatin and BLZ945 led to shift of macrophage phenotype from M2 to tumor suppressive M1	[166]
Ovarian	2D direct and indirect (conditioned medium)	A2780 OC8 NIH: OVCAR3 TOV21G	cisplatin carboplatin oxaliplatin	CAF derived glutathione and cysteine	IFNγ found in conditioned medium of activated CD8^+^T-cells, by altering glutathione and cysteine metabolism in fibroblasts	[188]
	2D indirect (conditioned medium)		cisplatin	CAF-derived CCL5 led to increased phosphorylation of STAT3 and Akt, promoting cell survival and proliferation	-	[189]
	2D direct with endothelial cells	Endothelial: HUVEC	lenvatinib	Lenvatinib was shown to reduce cord formation of endothelial cells *in vitro*, whereas inclusion of CAFs protects endothelial cells from its effect, therefore contributing to increased angiogenesis in tumor	-	[87]
	3D direct Patient-derived spheroids	Patient specimen	carboplatin	Cancer-derived PDGFβ activates fibroblasts which in turn express and activate HIF-1α related pathways	ripretinib – PDGF signaling inhibitor	[105]
PDAC	3D spheroid Direct	Bxpc3	cetuximab	CAF-derived EGF activates Akt pathway, promoting cell survival and proliferation	Effectivity of IGF1R inhibition suggested that drug combination has the potential to overcome observed resistance	[164]
	3D organoid Direct	Patient samples	gemcitabine	EMT induction; impaired drug delivery through ECM deposition	Collagenase, partially by ATRA through normalization of CAFs	[167]
Prostate	2D indirect (conditioned medium and transwell)	LNCaP, 22Rv1	doxorubicin, paclitaxel, mitomycin C	CAF derived glutathione prevents accumulation of the drug in cancer cells, attenuates drug-induced DNA damage and p53 induction, induces ROS production	-	[95]

## Conclusions

Cancer-associated fibroblasts have emerged as critical components of the tumor microenvironment, affecting disease progression through a plethora of mechanisms. They are a heavily heterogeneous population not only across various tissues, but also intra-tumoraly. Classifying these populations still remains challenging, however, it has become simpler with the more widespread use of novel technologies such as scRNAseq. Although transcriptomics data provide a great start for the classification of CAFs, more careful functional approach might be required to accurately describe their role.

Many of the biomarkers were reported to be used to distinguish between these subpopulations. Some of the markers can be found in a broader range of cancers, such as FAP or ACTA2, whereas some, such as COL16A1 in ccRCC, can be more tissue-specific. Although functionally several subtypes exist across tumors, the set of markers used to describe them can vary. Moreover, high expression of these biomarkers is often associated with either better or worse patient survival. Thanks to broadly available data from portals such as Human Protein Atlas, we can study these correlations across various cancers.

We acknowledge that this classification, based on myCAF/iCAF framework, primarily reflects a phenotypic and transcriptional state. It may very well be that these populations are not functionally monolithic. For instance, the myCAF population we describe, typically associated with dense desmoplasia, may contain subsets with tumor-restraining capabilities, as has been suggested in certain contexts [98]. Conversely, iCAFs, known for their inflammatory secretome, likely include subsets that potently drive tumor progression through paracrine signaling. The functional outcome of these CAF-tumor interactions is therefore highly context-dependent, influenced by factors such as tumor type, stage, and the presence of other immune components. Future work using single-cell transcriptomics and functional co-culture assays will be essential to deconvolute the specific tumor-promoting versus tumor-suppressive functions within the CAF subsets we have identified.

Apart from providing potential prognostic value in patients’ survival, CAFs have shown promise in predicting treatment resistance. They have been linked with resistance to chemo- and targeted therapies as well as to immune-checkpoint inhibitors. Various underlying mechanisms have already been proposed, amongst which we can distinguish CAF-mediated metabolic reprogramming of cancer cells or the creation of a physical barrier by excessive ECM deposition, limiting the availability of the compound. Therefore, CAFs became a promising therapeutic target, potentially reversing their induced drug resistance. However, limited CAF-targeted therapies exist, although in recent years, many more are undergoing clinical trials, especially in the context of PDAC. We hypothesize that shortly, the number of clinical studies assessing the efficacy of CAF-targeting treatment will increase as more studies highlight their impact on treatment resistance.

Despite growing understanding of roles of the stromal compartment, the majority of the pre-clinical models often miss the TME component. However, as underlined in this review, CAF-driven resistance should be considered as a big impact on the treatment development process. This, combined with high heterogeneity of CAFs between various cancers, underlines the necessity to include them in *in vitro* models for a better prediction of treatment response. In many of the described studies, CAFs manage to affect cancer cells in a similar way as they do *in vivo*, promoting tumor growth, contributing to increased migratory capabilities, affecting angiogenesis, and affecting treatment response. Though some effects were already observed in simplified 2D models, an increase in complexity resulted in a more accurate representation of the tumor. However, certain limitations still exist. Although essential for modelling stromal interactions, co-culture systems possess two limitations for studying CAF-mediated resistance, i.e., (i) they cannot ultimately attribute all observed effects to CAFs, and (ii) they cannot disentangle CAF-autonomous functions from the complex networks to stroma-cancer interactions. Moreover, CAFs interact with other cellular compartments of the TME, and only a handful of studies included them in their models, such as endothelial cells or selected immune cell populations. Furthermore, often these models are used in a static manner and under atmospheric oxygen levels. Inclusion of these models into microfluidic systems, in tumor-relevant oxygen levels, can contribute further to obtaining a more accurate tumor model. Moreover, when attempting to overcome CAF-induced resistance, screening of new compounds or new drug combinations should be expanded by assessing toxicity in healthy organ models [196]. Therefore, more studies are still required in various tumors to obtain a much better clinically relevant *in vitro* model.

## Data Availability

All data generated or analyzed during this study are included in this published article.

## Abbreviations

ACTA2/αSMA: α-smooth muscle actin
ATRA: Alltransretinoic acid
CAFs: Cancerassociated fibroblasts
ccRCC: Clear cell renal cell carcinoma
chRCC: Chromophobe renal cell carcinoma
CM: Conditioned media
COL: Collagen
CRC: Colorectal cancer
CSC: Cancer stem cells
ECs: Endothelial cells
ECM: Extracellular matrix
EMT: Epithelialtomesenchymal transition
FAP: Fibroblast Activation Protein
FGFR: Fibroblast growth factor receptor
GSH: Glutathione
HIF: Hypoxiainducible factor
IL: Interleukin
INF: Interface fibroblasts
LIF: Leukemia inhibitory factor
NF: Normal fibroblast
OS: Overall survival
PDAC: Pancreatic ductal adenocarcinoma
PDGF: Plateletderived growth factor
PFS: Progressionfree survival
pRCC: Papillary renal cell carcinoma
PSC: Pancreatic stellate cells
RCC: Renal cell carcinoma
SDF/CXCL: Stromal cell-derived factor
TGF: β-transforming growth factorβ
TME: Tumor microenvironment
VEGF: Vascular endothelial growth factor
